# The impact of life-long strength versus endurance training on muscle fiber morphology and phenotype composition in older men

**DOI:** 10.1152/japplphysiol.00208.2023

**Published:** 2023-10-26

**Authors:** Tiril Tøien, Jakob Lindberg Nielsen, Ole Kristian Berg, Mathias Forsberg Brobakken, Stian Kwak Nyberg, Lars Espedal, Thomas Malmo, Ulrik Frandsen, Per Aagaard, Eivind Wang

**Affiliations:** ^1^Department of Health and Social Sciences, Molde University College, Molde, Norway; ^2^Department of Sports Science and Clinical Biomechanics, Research Unit for Muscle Physiology and Biomechanics, University of Southern Denmark, Odense, Denmark; ^3^Department of Psychosis and Rehabilitation, Psychiatry Clinic, St. Olavs University Hospital, Trondheim, Norway; ^4^Department of Anesthesiology and Intensive Care, Drammen Hospital, Vestre Viken Hospital Trust, Drammen, Norway; ^5^Norwegian Defence University College, Norwegian Armed Forces, Oslo, Norway

**Keywords:** denervation, fast-twitch type II fibers, neural cell adhesion molecule (NCAM), nuclear clumps, older adults

## Abstract

Aging is typically associated with decreased muscle strength and rate of force development (RFD), partly explained by motor unit remodeling due to denervation, and subsequent loss of fast-twitch type II myofibers. Exercise is commonly advocated to counteract this detrimental loss. However, it is unclear how life-long strength versus endurance training may differentially affect markers of denervation and reinnervation of skeletal myofibers and, in turn, affect the proportion and morphology of fast-twitch type II musculature. Thus, we compared fiber type distribution, fiber type grouping, and the prevalence of atrophic myofibers (≤1,494 µm^2^) in strength-trained (OS) versus endurance-trained (OE) master athletes and compared the results to recreationally active older adults (all >70 yr, OC) and young habitually active references (<30 yr, YC). Immunofluorescent stainings were performed on biopsy samples from vastus lateralis, along with leg press maximal strength and RFD measurements. OS demonstrated similar type II fiber distribution (OS: 52.0 ± 16.4%; YC: 51.1 ± 14.4%), fiber type grouping, maximal strength (OS: 170.0 ± 18.9 kg, YC: 151.0 ± 24.4 kg), and RFD (OS: 3,993 ± 894 N·s^−1^, YC: 3,470 ± 1,394 N·s^−1^) as young, and absence of atrophic myofibers (OS: 0.2 ± 0.7%; YC: 0.1 ± 0.4%). In contrast, OE and OC exhibited more atrophic fibers (OE: 1.2 ± 1.0%; OC: 1.1 ± 1.4%), more grouped fibers, and smaller proportion of type II fibers (OE: 39.3 ± 11.9%; OC: 35.0 ± 12.4%) than OS and YC (all *P* < 0.05). In conclusion, strength-trained master athletes were characterized by similar muscle morphology as young, which was not the case for recreationally active or endurance-trained old. These results indicate that strength training may preserve type II fibers with advancing age in older men, likely as a result of chronic use of high contractile force generation.

**NEW & NOTEWORTHY** Aging is associated with loss of fast-twitch type II myofibers, motor unit remodeling, and grouping of myofibers. This study reveals, for the first time, that strength training preserves neural innervation of type II fibers, resulting in similar myofiber type distribution and grouping in life-long strength-trained master athletes as young moderately active adults. In contrast, life-long endurance-trained master athletes and recreationally active old adults demonstrated higher proportion of type I fibers accompanied by more marked grouping of type I myofibers, and more atrophic fibers compared with strength-trained master athletes and young individuals. Thus, strength training should be utilized as a training modality for preservation of fast-twitch musculature, maximal muscle strength, and rapid force capacity (RFD) with advancing age.

## Graphical abstract

**Figure:**
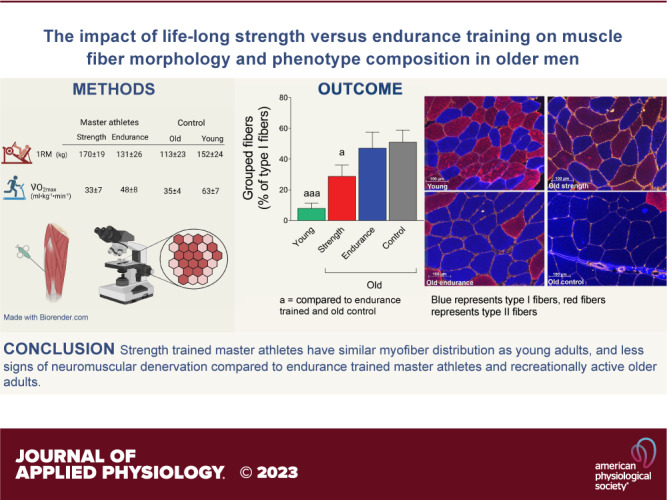

## INTRODUCTION

The maximal strength and rapid force capacity (rate of force development; RFD) of human skeletal muscle typically decline with age (1–4). One of the main contributors to this decline is the peripheral denervation, and subsequent loss of myofibers (5–7) accompanied by a preferential atrophy of fast-twitch type II fibers (1, 8, 9). Reinnervation and remodeling of motor units may occur as a compensatory response to age-related denervation (10, 11), potentially causing fiber type changes and leading to signs of fiber type grouping (12). There appears to be more pronounced grouping of type I fibers (11, 13), due to age-related denervation of type II fibers followed by reinnervation of part of the denervated myofiber pool by low-threshold motor units that originally innervate type I fibers, resulting in a higher proportion of type I fibers and grouping of these fibers (14).

Although the decline in maximal muscle strength with age appears to be inevitable, it may be decelerated by physical exercise (15). As a testament to successful aging, older female master athletes, consisting of both endurance- and strength-trained athletes, have been characterized by signs of attenuated neurogenic atrophy and superior reinnervating capacity compared with their frail age-matched counterparts (11). This was manifested as fewer nuclear clumps, a smaller decline in fiber type IIa/I size ratio, and less variable intersubject accumulation of neural cell adhesion molecule (NCAM)-positive fibers and atrophic fibers in master athletes compared with frail controls (11). NCAM is recognized as a marker of denervation (16, 17), but may also be upregulated during reinnervation (16), whereas the presence of atrophic fibers (i.e., very small fibers) and nuclear clumps may reflect neurogenic atrophy (18–20) with the latter detectable several years after complete upper motor neuron injury (18). There are also indications that life-long training may prevent the loss of motor units, as track and field master athletes had a higher number of estimated motor units than older untrained participants (21, 22). However, this has not been a universal finding, as similar motor unit numbers have been observed in endurance- and strength-trained master athletes compared with untrained, older controls (10, 23). This discrepancy may be explained by methodological differences regarding the methods used to estimate motor units (electrophysiological estimates; 24). Moreover, investigations of life-long training in master athletes typically do not distinguish between training modes, thus it remains elusive whether specific exercise modalities (i.e., endurance vs. strength training) may provide more protective effects to prevent the age-related denervation of myofibers.

We have previously documented that maximal muscle strength and RFD were higher in 70-yr-old life-long strength-trained master athletes compared with moderately active young (20–30 yr) individuals (3). Moreover, the high muscle strength levels observed in strength-trained master athletes is accompanied by a superior descending neural drive (elevated evoked V-wave responses) to maximally contracting myofibers, when compared with sedentary and recreationally active age-matched individuals (25), indicating that strength training may be particularly beneficial for maintaining recruitment and in turn innervation of fast-twitch type II fibers. In contrast, endurance-trained master athletes appear to be characterized by different neural pathways, dissociated from the activation of type II fibers and the ability to perform very strong muscle contractions (26). In line with this notion, life-long strength-trained master athletes have been documented to have significantly larger type IIa and IIx fiber areas, respectively, compared with life-long endurance-trained master athletes as well as sedentary age-matched older adults (27), further suggesting that long-term strength training may offer significant benefits compared with other exercise modalities, for the preservation of type II fiber morphology with increasing age.

There is mounting evidence that strength training may be imperative for the maintenance of maximal force-generating capacity with advancing age. However, markers of denervation and fiber type grouping have not previously been investigated along with the assessment of myofiber size and phenotype composition in chronically strength-trained versus endurance-trained master athletes. Thus, the aim of the present study was to investigate if life-long trained master athletes exhibit different patterns of myofiber distribution, fiber type grouping, and myofiber atrophy, or differential signs of denervation and reinnervation depending on their chronic training background (strength vs. endurance), and to make similar comparisons to young and older habitually active adults. Specifically, it was hypothesized that master athletes engaged in life-long strength training would demonstrate maintenance of type II fiber distribution and attenuated or absent signs of myocellular denervation (muscle fiber grouping, atrophic fibers, and number of nuclei clumps) and muscle regeneration (NCAM-positive fibers), respectively, compared with age-matched life-long endurance-trained master athletes, and recreationally active older individuals. Further, we hypothesized that young habitually active adults would exhibit fewer signs of myofiber denervation and regeneration compared with aged adults irrespective of their training status.

## MATERIALS AND METHODS

### Participant Characteristics

A total of 42 men were included in the study. Participants (>65 yr) were recruited based on their physical training status, and are also part of another study (26). Strength-trained master athletes (OS, 73 ± 4 yr, *n* = 10) were recruited from local powerlifting and weightlifting clubs, whereas endurance-trained master athletes were recruited from local Track and Field clubs (OE, 72 ± 6 yr, *n* = 8) limited to athletes participating in endurance events of long duration (≥3,000 m). Participants were considered athletes if they had been actively training and competing over the past 2 years in their respective sport. Master athletes had trained systematically most of their lives, although they reported some sporadic breaks throughout their careers. As such, they are considered life-long trained athletes. No female strength-trained master athletes could be identified in the age groups we aimed to recruit, despite our best efforts to find female master athletes engaged in strength sports by searching in data bases and results from past competitions at regional and national levels. Half of the endurance-trained master athletes reported performing some general strength exercises <1 session/wk. This was typically performed as circuit training with a focus on core muscles. In contrast, the strength-trained master athletes reported mostly heavy strength training performed with few repetitions (typically 5 reps or less) using heavy exercise loads (>80% of one repetition maximum; 1RM) in squat, deadlift, snatch, clean and jerk, and bench press. Strength-trained master athletes also reported some sporadic endurance-based activities, such as swimming, cycling, and going for walks. Recreationally active control participants, engaging in activities like hiking and skiing (OC, 75 ± 6 yr, *n* = 13) were recruited from senior societies, and finally moderately active young references (YC, 25 ± 4 yr, *n* = 11) were recruited among local University students. The activity level of recreationally active older and young was confirmed using the International Physical Activity Questionnaire (IPAQ). Exclusion criteria included: documented history of neurological, musculoskeletal, cardiovascular disease, and/or pulmonary disease. The study was approved by the Regional Ethics Committee (REC number: 2018/1207) and conducted in accordance with the Declaration of Helsinki. All subjects gave their written informed consent before inclusion in the study. Subject characteristics are summarized in Table 1.

**Table 1.** Subject characteristics

		Old		
	Young (*n* = 11)	Strength (*n* = 10)	Endurance (*n* = 8)	Control (*n* = 13)
Body mass, kg	76.0 ± 8.9^e^	85.9 ± 12.1	69.0 ± 8.1^d^	83.5 ± 9.6
Body height, cm	180 ± 6	174 ± 6	175 ± 8	179 ± 7
Thigh muscle volume, cm^3^	5,259 ± 950	5,199 ± 1,418	4,657 ± 860	4,863 ± 942
V̇o_2max_, mL·kg^−1^·min^−1^	63.1 ± 6.6^ccc^	33.2 ± 6.8	47.5 ± 8.1^ddd^	35.2 ± 3.8
V̇o_2max_, L·min^−1^	4.79 ± 0.65^ccc^	2.84 ± 0.61	3.24 ± 0.48	2.94 ± 0.46

Group means ± SD. ^c^Different from all older adults. ^d^Different from older strength athletes and older controls. ^e^Different from older strength athletes. One and three symbols indicate significance level of *P* ≤ 0.05 and 0.001, respectively. V̇o_2max_, maximal oxygen uptake.

### Study Outline

Participants performed all physical testing procedures on a single day. On arrival in the laboratory, subjects were weighed, and anthropometric measurements were obtained. Subjects proceeded to physical testing, where familiarization and warm up procedures were combined and instructions on how to perform each individual test were provided. 1RM muscle strength and RFD were measured in an instrumented leg press apparatus (25, 28) followed by measurement of maximal oxygen uptake (V̇o_2max_) during walking or running on a treadmill. Subjects were also asked to answer a questionnaire about current or previous steroid and growth hormone usage. Biopsies were obtained from the vastus lateralis (VL) 10–14 days after the physical tests.

### Anthropometry

Thigh muscle volume was estimated using skinfold caliper recordings to the nearest 1 mm (Baty International) using following equation, previously described, and validated against magnetic resonance imaging (MRI; 29):

(*1*) $${\mathrm{Vol}}_{\text{anthropo}}=\left(L/12\pi \right)\cdot \left(C1^{2}+C2^{2}+C3^{2}\right)-\left[\left(S-0.4\right)/2\right]\cdot \;L\cdot \left[\left(C1+C2+C3\right)/3\right],$$

where *L* is thigh length, *S* is skinfold thickness, and *C* is circumference. All measurements were conducted three times at each site (*C*1, *C*2, and *C*3), and the average value was used for analysis. Thigh length was measured from the greater trochanter of the femoral head to the lateral femoral epicondyle. Thigh circumference was measured at the mid-point of the thigh length (*C*2) and 10 cm proximal (*C*1), and distal (*C*3) to the midpoint. Skinfold measurements were performed at the midpoint laterally, medially, and anteriorly. Subsequently, muscle volume was estimated using the following equation (29):

(*2*) $$\text{Thigh muscle volume }\left({\text{cm}}^{3}\right)=0.866\cdot {\text{Vol}}_{\text{anthropo}}-1,750.$$

### Maximal Muscle Strength and Rate of Force Development

1RM strength of the leg extensors was measured using a horizontal leg press apparatus instrumented with a force plate and movable weight (Technogym silver line, Gambettola, Italy), as previously described (28). The subjects performed three warm-up sets of eight down to two repetitions with increasing loads up to ∼60% of expected 1RM, before commencing the 1RM test. Instructions were given to the participants to lower the weight slowly (starting from near 180° knee flexion) and have a short pause (∼1 s) at the bottom of the movement (at ∼90° knee flexion) before maximally contracting their lower limb muscles to move the load as forcefully as possible back to its starting position. These instructions were given already during warm-up and repeated for each attempt throughout the 1RM strength test. The load was gradually increased by 5–20 kg if the participant was able to lift the load successfully, and 1RM typically was reached within five attempts. Each attempt was separated by ∼3 min of rest. The highest weight successfully lifted according to the instructions was recorded as 1RM strength.

A piezoelectric force plate was used to record dynamic RFD in the same leg extensor apparatus, where the orthogonal reaction force under the feet was digitally sampled at 2 kHz (model 9286AA; Kistler, Winterthur, Switzerland). The test load was set to the closest 10 kg level matching or above the subject’s body weight in accordance with previous procedures (25). Instructions during the RFD measurements were similar to those given during 1RM testing, emphasizing a slow, controlled eccentric movement followed by a marked stop at the bottom of the movement (∼1 s), before initiating a concentric movement at maximal intentional speed. The highest RFD out of three trials was selected for analysis. RFD (ΔF/Δ*t*) was calculated as the rise in force (ΔF) divided by the duration of the time interval (Δ*t*) between 10% and 90% peak concentric force (30).

### Maximal Oxygen Uptake

Participants walked at 4.5 km·h^−1^ at 5% inclination for 5 min to warm up before the treadmill (Woodway, Waukesha, WI) speed and/or inclination was gradually increased every 3 min to reach V̇o_2max_ (31). Pulmonary gas exchange variables were obtained every 10 s (Metamax II, Cortex Biophysik GmbH, Leipzig, Germany). The highest 30-s average of V̇o_2_ was reported as V̇o_2max_. V̇o_2max_ was accepted when no further increase in V̇o_2_ was observed despite increased speed/inclination and a respiratory exchange ratio ≥ 1.05 was present.

### Muscle Biopsy Sampling

About 10–14 days following physical testing in all groups, muscle biopsies were obtained from a depth of ∼3.5 cm from the middle portion of the vastus lateralis, slightly distal to the ventral midline of the muscle (32, 33). Participants were asked to refrain from alcohol and strenuous exercise within 48 h leading up to the biopsy. A 6-mm Bergström needle attached to a suction syringe was used after injecting local xylocaine (1%) anesthesia at the superficial level of the muscle fascia. Following removal, the tissue samples were aligned and mounted in Tissue-Tek (4583, Sakura Finetek, AV Alphen aan den Rijn, the Netherlands) and subsequently frozen in isopentane precooled in liquid nitrogen and stored in a −80°C freezer until further analyses. Before analysis, transverse serial sections (8 μm) of the embedded muscle biopsy specimen were cut at −22°C using a cryostat (HM560; Microm, Walldorf, Germany) and were mounted on glass slides.

### Immunofluorescence Staining Procedure and Analysis

To visualize NCAM-positive fibers, cryosections were mounted in a 4% formaldehyde fixation solution [Triton X-100 (10%), formaldehyde (37%), and phosphate-buffered saline (PBS, ×10)] for 10 min, whereas sections for visualization of fiber types (I, IIa, and IIx) and the myofiber perimeter was not. Subsequently, for all sections, a wash procedure (3 × 3 min in PBS, ×10) was completed before blocking (Vector SP6000) for 10 min. Next, NCAM staining was performed using a two-step protocol consisting of anti-NCAM (DAKO, 1:100, M7304) and anti-Laminin (DAKO, 1:1,000, Z0097) incubation followed by MHC-slow [BA-F8, 1:50, Developmental Studies Hybridoma Bank (DSHB)] using the following secondary antibodies (A21424, A21206, and A21140, ThermoFisher). For visualization of fiber types (I, IIa, and IIx) and myofiber membrane perimeter, sections were stained anti-MHC-slow, IIa and IIx and anti-laminin (BA-F8 1:50, SC-71 1:250, 6H1 1:25; DSHB and Z0097 1:1,000; DAKO), respectively, followed by a secondary antibody step (A21240, A21140, AB_2535711, and A21206; ThermoFisher). All primary and secondary antibody steps were incubated for 60 min at room temperature, separated by wash procedures. Finally, all stained sections were mounted with mounting medium (H5501; Vector). Immunofluorescence images were captured using a microscope (Carl Zeiss Axio Imager M1, Germany) and a high-resolution AxioCam (Carl Zeiss). All images were obtained using the ×10 objective and between image standardized exposures.

**Figure 1.**
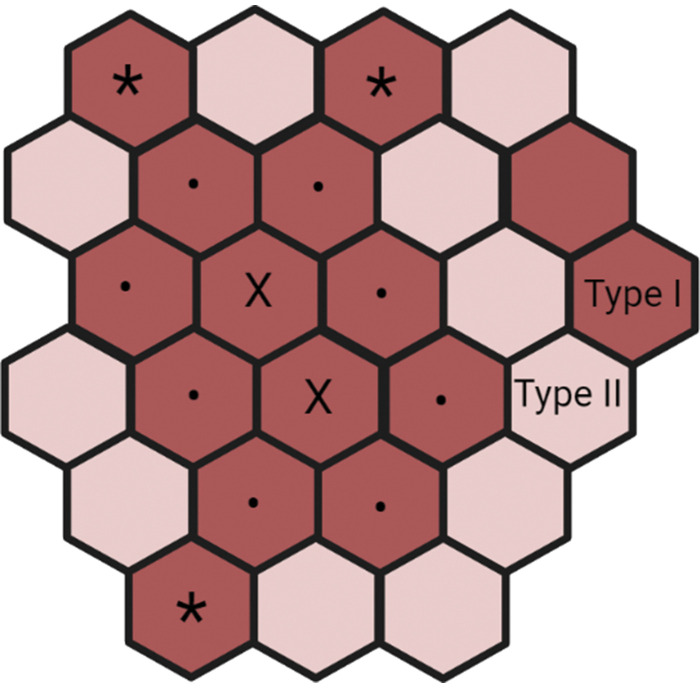
A schematic visualization of fiber type grouping variables. Deep red represents type I fibers and light red represents type II fibers. X indicates enclosed type I fibers, • indicates enclosing type I fibers, *remaining (i.e., contiguous with the enclosing fibers, not separated by a fascicle) type I fibers. All enclosed, enclosing, and remaining fibers were counted as one group. The number of fibers type groups was expressed per 1,000 fibers. The number of enclosed and enclosing fibers were presented as a percentage of type I fibers. The total number of grouped fibers (total number of enclosed + enclosing + remaining fibers for each subject) was presented as percentage of type I fibers and percentage of whole fiber type count. Group size was presented as number of fibers per group. Created in Biorender.com.

All analyses were performed using a digital analysis program (Carl Zeiss, AxioVision 4.6). Type I (blue), type IIa (red), and type IIx (green) myofibers were identified and analyzed for fiber number and cross-sectional area. Myofiber distribution was expressed as proportion of total fiber number and proportion of mean fiber area (area-weighted fiber distribution). The method of Jennekens et al. (34) was used to quantify fiber type grouping of type I fibers in given cross sections. An “enclosed fiber” was defined as a type I muscle fiber surrounded by only type I fibers. A “fiber type group” was defined as a group of fibers with at least one enclosed fiber (35). The number of enclosed fibers was counted manually, along with number of enclosing fibers (a fiber that surrounded an enclosed fiber) and remaining fibers [fibers that were neither enclosed or enclosing fibers, but contiguous with the enclosing fibers and not separated by a visible fascicle (14); Fig. 1]. The number of fiber type groups was expressed per 1,000 fibers. The number of enclosed and enclosing fibers was presented as a percentage of type I fibers, and the total number of grouped fibers (enclosed + enclosing + remaining fibers) was presented as percentage of type I fibers and percentage of whole fiber type count. Group size was presented as number of fibers per group. Atrophied fibers were identified as the mean fiber area represented by the lowest 1st percentile in YC following analysis of all fibers in this group, as previously used (11, 36), which corresponded to fibers with a size ≤1,494 µm^2^. Previous findings suggest that fibers of this size are atrophied, as >90% expressed Nav_1.5_, a marker of denervation (20). On average, 867 ± 471 myofibers (means ± SD) were analyzed per biopsy for the assessment fiber type distribution, 611 ± 348 for grouping and 160 ± 111 for fiber area.

NCAM-positive myofibers were identified as fibers with a NCAM expression exceeding 2 SD above the mean expression of two clearly negative fibers. NCAM-positive fibers were reported per 1,000 fibers. Moreover, the percentage of NCAM-positive fibers coexpressing MHC I was calculated, whereas the mean fiber area of NCAM-positive fibers was determined and compared with the average area of type I and type II fibers identified in the analyses of all fibers. Only subjects with NCAM-positive fibers were included in the latter analyses. An average 667 ± 197 myofibers were analyzed per biopsy sample for NCAM.

All fiber type and grouping analyses were performed by the same assessor, who was blinded to subject ID and group adherence.

### Histochemistry Staining Procedure and Analysis

Hematoxylin and eosin (H&E) were used to stain myofiber cross sections to assess nuclear clumps. The presence of nuclear clumps (nuclear bags) were used as a marker of long-term neurogenic atrophy (11, 19).

H&E images were captured using a microscope (Nikon TI Eclipse Widefield) and a high-resolution camera (sCMOS, Oxford Instruments). All analysis was performed using AxioVision. Nuclear clumps were counted by an assessor who was blinded in terms of subject ID and group. On average 335 ± 74 fibers were analyzed per biopsy sample.

### Statistical Analysis

Normality (Gaussian distribution) was assessed using the Kolmogorov–Smirnov test and by visual inspection of Q-Q plots after removal of outliers. Outliers were identified by examining box plots and histograms and removed if outside the 3rd quartile + 1.5 × interquartile range and 1st quartile − 1.5 × interquartile range (37). 1RM, RFD, V̇o_2max_, fiber type data, and NCAM-positive fibers coexpressing MHC I were found to follow a normal distribution. As expected, grouping variables in YC did not follow normal distribution, as there were many individuals without grouping. However, since they were used as a young reference group relative to the older subject groups (all of whom were normally distributed), a parametric test was used. As such, a one-way ANOVA was used to detect between-group interactions, followed by Fisher’s LSD to investigate differences between groups. The number of nuclear clumps, NCAM-positive fibers, and atrophic fibers did not follow normal distribution. Consequently, the Kruskal–Wallis test was used to detect between-group differences, followed by Mann–Whitney *U* test between groups (38). The area of NCAM-positive fibers was compared with the average area of all fibers (type I and type II combined) using paired samples *t* test, as these data adhered to a normal distribution. Significance level was *P* ≤ 0.05 (two-tailed), and borderline effects were presented when *P* ≤ 0.10.

## RESULTS

None of the participants reported any previous or current intake of growth hormones, testosterone, or related anabolic substances. One outlier in YC was identified in all grouping variables except group size. Similarly, one outlier in OS was identified in all grouping variables except group size and number of grouped fibers (% type I fibers). Further, one outlier from OC was removed from the analysis of number of groups per 1,000 fibers, and one outlier from OE was removed from the group size analysis. Two outliers were removed from the analysis of NCAM-positive fibers in OC and one from OE, whereas four outliers were removed from the analysis of atrophied myofibers (one outlier in each group). Nuclear clumps content was not analyzed in one participant in OS as well as one participant in OE due to ice-crystal artifacts in the biopsy material.

### Maximal Strength and Rate of Force Development

**Figure 2.**
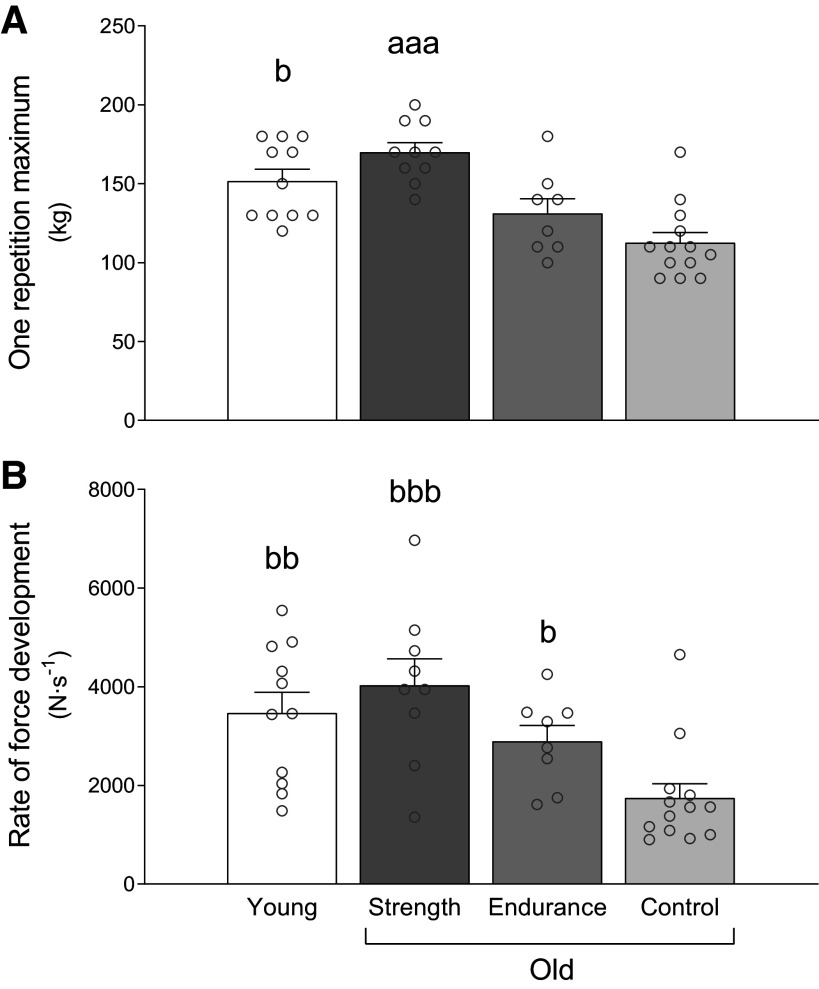
One repetition maximum (*A*) and dynamic leg press rate of force development (*B*) in young recreationally active (*n* = 11), older strength athletes (*n* = 10), older endurance athletes (*n* = 8), and older recreationally active control participants (*n* = 13). Data are presented as means ± SE and individual responses. ^a^Different from older endurance athletes and older control participants, ^b^different from older control participants. One, two, and three symbols indicate significance level of *P* ≤ 0.05, 0.01, and 0.001, respectively. The statistical test used was one-way ANOVA.

Leg press 1RM strength differed between subject groups (main effect *P* < 0.001, Fig. 2*A*), where OS demonstrated higher 1RM than OC and OE (both *P* < 0.001) while tending to be higher than YC (*P* = 0.080). OE tended to have higher 1RM strength than OC (*P* = 0.083), whereas YC showed higher 1RM than OC (*P* < 0.001) and tended to show higher 1RM than OE (*P* = 0.064).

A main effect for RFD also was observed (*P* < 0.001, Fig. 2*B*), revealing OS to have higher leg press RFD than OC (*P* < 0.001) and tended to have higher RFD than OE (*P* = 0.072). There was no difference detected between YC and OS (*P* = 0.329). OE had a higher RFD than OC (*P* = 0.049). Finally, there was no difference between YC and OE (*P* = 0.334), whereas OC demonstrated lower RFD than YC (*P* = 0.002).

### Maximal Oxygen Uptake

Both relative (expressed relative to body mass) and absolute V̇o_2max_ differed between subject groups (main effect *P* < 0.001; Table 1). Specifically, OE showed higher relative V̇o_2max_ than OS and OC (both *P* < 0.001). Likewise, YC demonstrated higher V̇o_2max_ than all older groups (all *P* < 0.001), whereas there was no difference between OS and OC (*P* = 0.469). YC also had higher absolute V̇o_2max_ than all older groups (*P* < 0.001), whereas there was no difference in absolute V̇o_2max_ between any of the older groups (*P* = 0.135–0.664).

### Muscle Fiber Distribution

Type II fiber distribution (% number of fibers) was higher in OS (52.0 ± 16.4%; main effect *P* = 0.012) compared with OC (35.0 ± 12.4%; *P* = 0.006) while tending to be higher than OE (39.3 ± 11.9%; *P* = 0.063). No difference in type II fiber distribution could be detected between OS and YC (51.1 ± 14.4%; *P* = 0.895). YC had higher type II fiber distribution than OC (*P* = 0.007) and borderline higher than OE (*P* = 0.075). Type I fiber distribution (% number of fibers) showed inverse trends compared with type II fibers (OS: 48.1 ± 16.4%, OE: 60.7 ± 11.9%, OC: 65.0 ± 12.4%, YC: 48.9 ± 14.4%).

Type IIa fiber distribution (% number of fibers) was higher in OS (41.4 ± 9.2%; main effect *P* = 0.008) compared with OC (29.7 ± 11.0%; *P* = 0.006) and borderline higher than OE (33.5 ± 6.5%; *P* = 0.088). No difference could be detected between OS and YC (41.9 ± 9.8%; *P* = 0.918). YC showed higher type IIa distribution (% number of fibers) than OC (*P* = 0.004) while tending to be higher than OE (*P* = 0.067). There was no main effect for the distribution of type IIx fibers across subject groups (OS: 10.5 ± 12.9%, OE: 5.9 ± 5.8%, OC: 5.3 ± 7.1%, YC: 9.3 ± 8.9%; *P* = 0.480).

**Figure 3.**
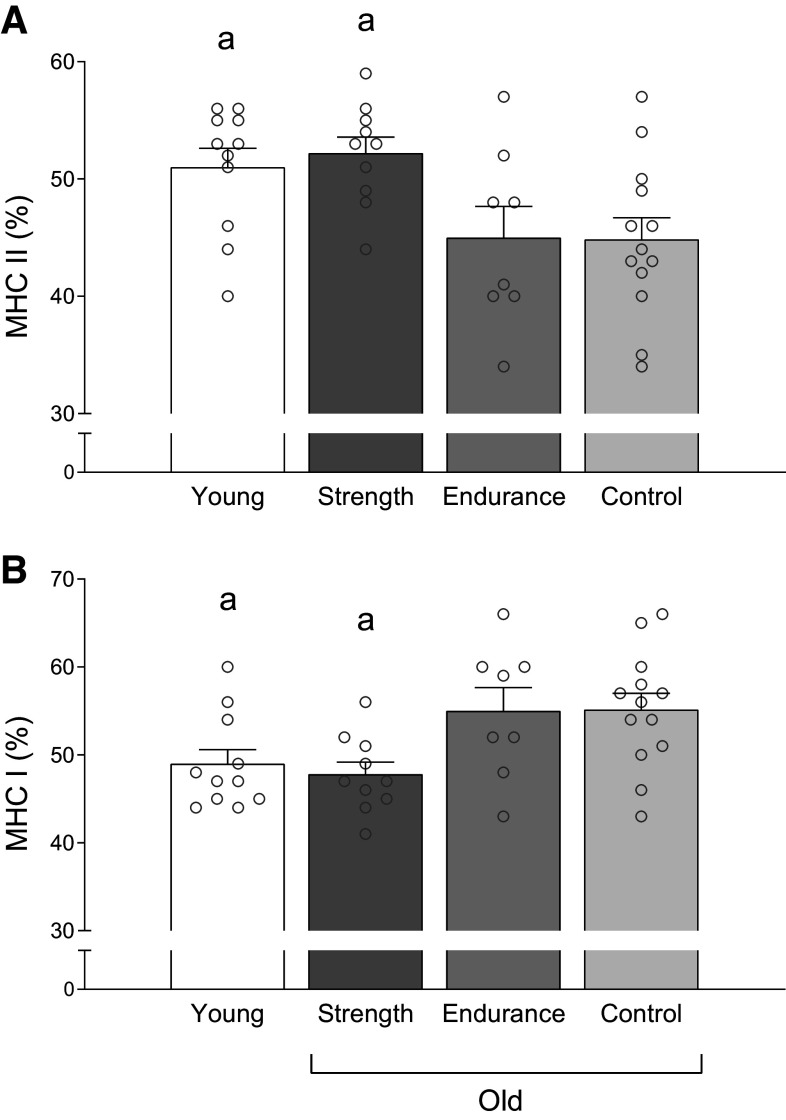
Area-weighted fiber distribution (% of mean fiber area) of type II (*A*) and type I fibers (*B*) in young recreationally active (*n* = 11), older strength athletes (*n* = 10), older endurance athletes (*n* = 8), and older recreationally active control participants (*n* = 13). Data are presented as means ± SE and individual responses. ^a^Different from older endurance athletes and older control participants. One symbol indicates a significant level of *P* ≤ 0.05. The statistical test used was one-way ANOVA. MHC, myosin heavy chain.

Area-weighted type II fiber distribution (% fiber area; main effect *P* = 0.010; Fig. 3) was higher in OS compared with OE (*P* = 0.016) and OC (*P* = 0.006). There was no difference detected between OS and YC (*P* = 0.652). YC had higher area-weighted type II fiber distribution than OC (*P* = 0.017) and OE (*P* = 0.039).

There was no main effect for mean cross-sectional area of type I fibers (*P* = 0.590), type II fibers (*P* = 0.140), type IIa fibers (*P* = 0.157), or type IIx fibers (*P* = 0.250; Table 2).

**Table 2.** Mean cross-sectional area of type I, II, IIa, and IIx myofibers from vastus lateralis, and number of nuclear clumps and NCAM-positive fibers per 1,000 fibers

		Old		
	Young	Strength	Endurance	Control
Type I, µm^2^	4,439 ± 1,135	4,932 ± 1,019	5,059 ± 848	5,039 ± 1,478
Type II, µm^2^	4,644 ± 1,204	5,353 ± 1,233	4,176 ± 1,148	4,146 ± 1,485
Type IIa, µm^2^	5,162 ± 1,335	5,602 ± 1,289	4,633 ± 1,253	4,427 ± 1,257
Type Iix, µm^2^	4,127 ± 1,127	5,159 ± 1,478	3,853 ± 1,197	3,837 ± 1,934
Nuclear clumps, per 1,000 fibers	0.0 ± 0.0^d^	1.9 ± 3.7	0.0 ± 0.0	3.6 ± 6.3
NCAM-positive, per 1,000 fibers	2.2 ± 2.3	7.3 ± 9.8	1.0 ± 1.4	7.4 ± 8.5

Group means ± SD. ^d^Different from older strength athletes and older control subjects; *P* < 0.05. NCAM, neural cell adhesion molecule.

### Muscle Fiber Grouping

**Figure 4.**
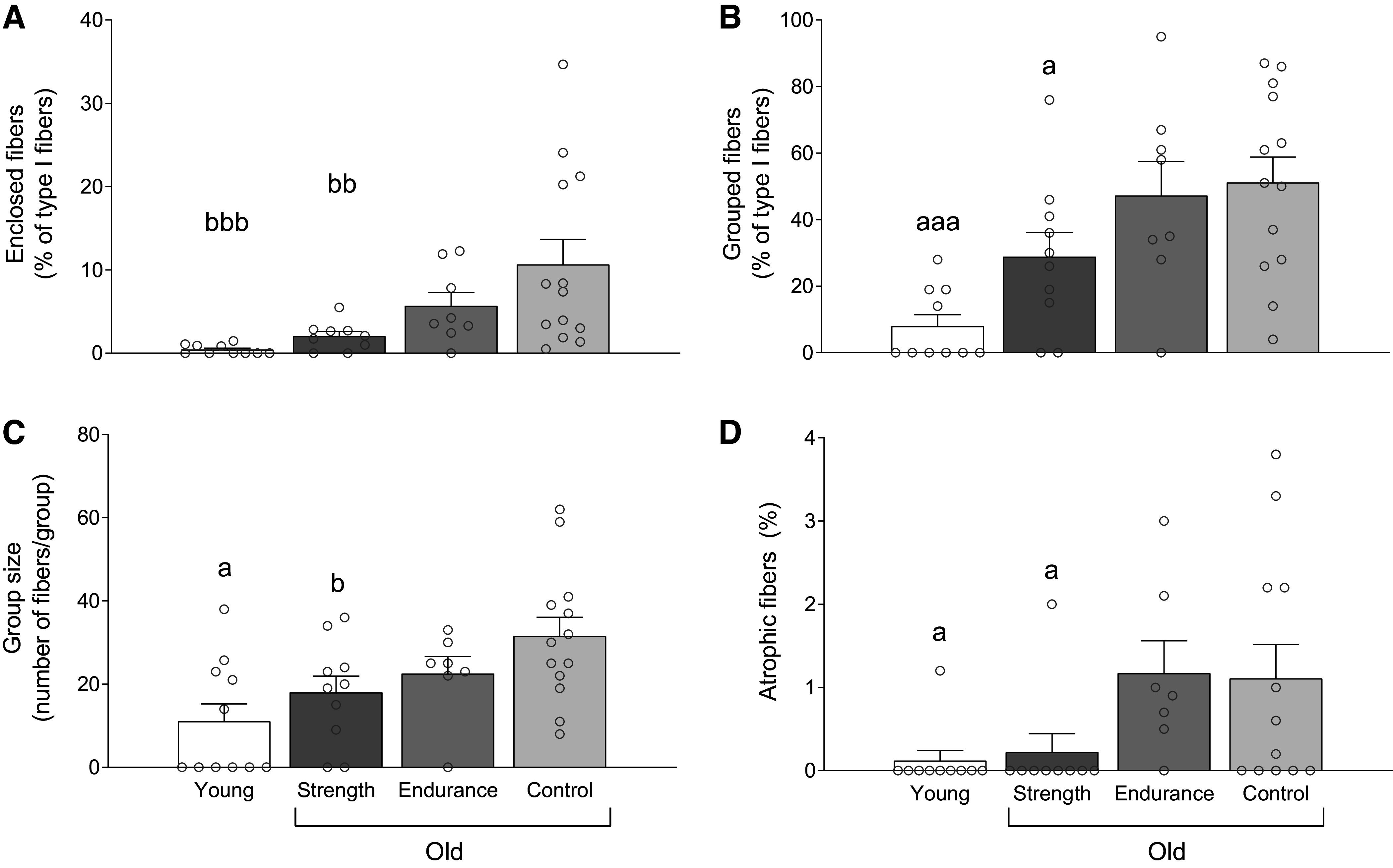
Enclosed fibers (percentage of type I fibers; *A*), grouped fibers (percentage of type I fibers; *B*), group size (number of fibers per group; *C*), and percentage of atrophic fibers (fibers < 1,494 µm^2^; *D*) in young recreationally active (*n* = 10 in *A*, *B*, and *D*; *n* = 11 in *C*), older strength athletes (*n* = 9 in *A* and *D*; *n* = 10 in *B* and *C*), older endurance athletes (*n* = 7 in *C* and *D*; *n* = 8 in *A* and *B*), and older recreationally active control participants (*n* = 12 in *D*; *n* = 13 in *A*–*C*). Data are presented as means ± SE and individual responses. ^a^Different from older endurance athletes and older control participants; ^b^different from older control participants. One, two, and three symbols indicate significance level of *P* ≤ 0.05, 0.01, and 0.001, respectively. One-way ANOVA was used in *A*–*C* and Kruskal–Wallis followed by Mann–Whitney *U* test in *D*.

**Figure 5.**
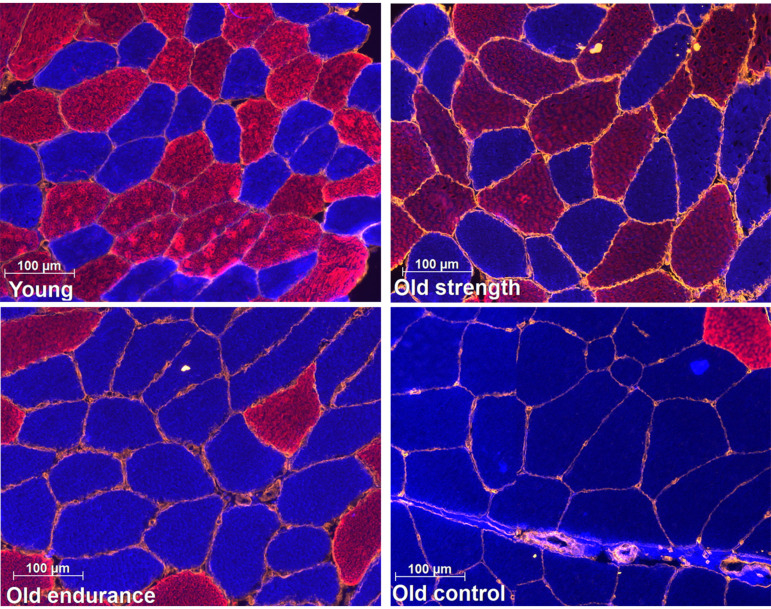
Representative images of fiber type distribution and type I grouping in young recreationally active, older strength athletes, older endurance athletes, and older recreationally active control participants. Blue color represents myosin heavy chain type I, red color represents myosin heavy chain type II and orange represents laminin.

The number of enclosed fibers (% of type I fibers; main effect *P* = 0.004; Fig. 4*A*) was lower in OS than OC (*P* = 0.005), but there was no difference between OS and OE (*P* = 0.265). No difference was detected between OS and YC (*P* = 0.595), OE and OC (*P* = 0.103), or OE and YC (*P* = 0.102), whereas OC had more enclosed fibers than YC (*P* < 0.001). Figure 5 displays representative images of fiber type distribution and myofiber grouping in each group.

There were fewer enclosing fibers (% of type I fibers; main effect *P* < 0.001) in OS (7.9 ± 5.2%) than OC (19.7 ± 11.5%; *P* = 0.004) and OE (17.2 ± 11.5%; *P* = 0.036), whereas no difference was noted between OE and OC (*P* = 0.526). No differences were observed between OS and YC (2.1 ± 2.8%; *P* = 0.168), whereas OE and OC both showed more enclosing fibers than YC (*P* < 0.001).

Likewise, the number of grouped fibers (% of type I fibers; main effect *P* < 0.001; Fig. 4*B*) was lower in OS than OC (*P* = 0.008) and OE (*P* = 0.039), whereas there was no difference between OE and OC (*P* = 0.703). There was a tendency for a difference was detected between OS and YC (*P* = 0.057), whereas OE and OC showed more grouped fibers than YC (*P* < 0.001).

Fewer grouped fibers (% of total fiber type count; main effect *P* < 0.001) were observed in OS (12.3 ± 9.3%) than OC (35.9 ± 23.4%; *P* = 0.004) and OE (31.4 ± 21.9%; *P* = 0.031), whereas there was no difference between OE and OC (*P* = 0.566). Likewise, no difference was detected between OS and YC (4.5 ± 6.3%; *P* = 0.335), whereas OE (*P* = 0.003) and OC (*P* < 0.001) had more grouped fibers than YC.

Group size (main effect *P* = 0.005; Fig. 4*C*) was smaller in OS compared with OC (*P* = 0.023) but not OE (*P* = 0.240). No difference was detected between OC and OE (*P* = 0.440) or between YC and OS (*P* = 0.248), whereas OE (*P* = 0.032) and OC (*P* < 0.001) had larger group size than YC.

Number of fiber type groups per 1,000 fibers (main effect *P* < 0.001) was lower in OS (5.6 ± 3.8) compared with OC (10.1 ± 5.2; *P* = 0.033) while tending to be lower than OE (9.7 ± 6.0; *P* = 0.073), but not different than YC (2.1 ± 2.7; *P* = 0.107). OC and OE had higher number of fiber groups than YC (*P* < 0.001) with no differences between OC and OE (*P* = 0.865).

### Atrophic Fibers

Atrophic fibers (fiber cross-sectional area <1,494 µm^2^) were detected in a total of 19 subjects (two in OS, two in YC, seven in OE, and eight in OC). There was a main effect (*P* = 0.006), revealing that OC (*P* = 0.034) and OE (*P* = 0.009) had higher percentage atrophic fibers than OS (Fig. 4*D*). A higher abundance of atrophic fibers also was observed in OC (*P* = 0.024) and OE (*P* = 0.005) compared with YC. In contrast, atrophic fiber content did not differ between OS and YC (*P* = 0.878) or OC and OE (*P* = 0.606).

### Denervation Markers

Nuclear clumps were detected in a total of eight subjects, three in OS and five in OC. A main effect was evident for nuclear clumps (*P* = 0.046; Table 2), where OS (*P* = 0.044) and OC (*P* = 0.025) showed higher content than YC, whereas there was no difference between YC and OE (*P* = 1.000). Also, there was no difference between OS and OC (*P* = 0.727), OS and OE (*P* = 0.103), however, a tendency emerged for higher count of nuclear clumps in OC than OE (*P* = 0.068).

NCAM-positive fibers were detected in a total of 27 subjects: 6 in YC and OS, 4 in OE, and 11 in OC. There was no main effect across subject groups in the number of NCAM-positive fibers (*P* = 0.097; Table 2).

**Figure 6.**
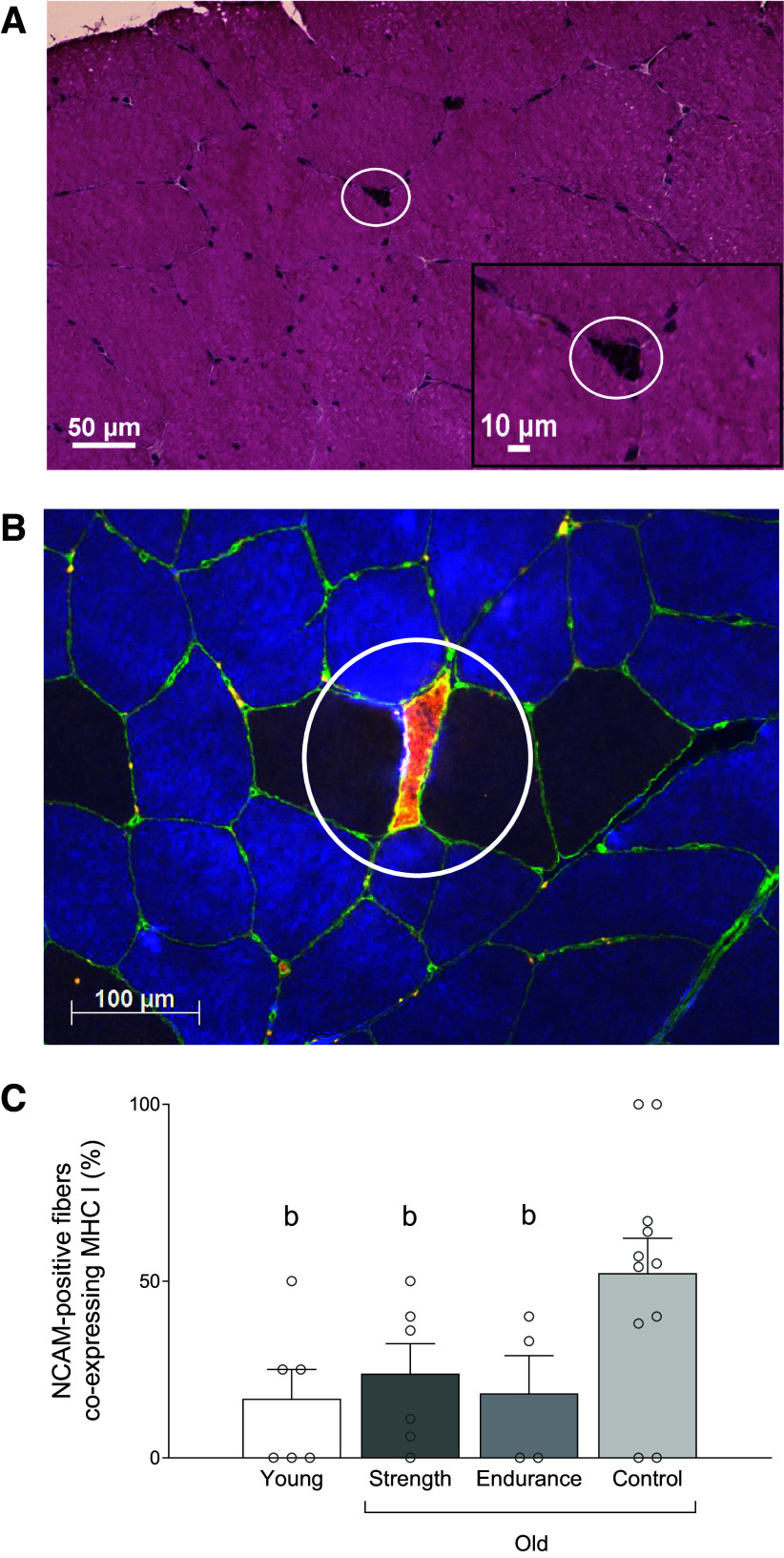
A representative image of nuclear clumps (indicated by circle) from hematoxylin and eosin-stain from older recreationally active participant (*A*), a representative image of neural cell adhesion molecules (NCAM)-positive fiber (indicated by circle) from one older recreationally active participant (*B*), and percentage of NCAM-positive fibers coexpressing myosin heavy chain (MHC) type I (*C*) in young control (*n* = 6), older strength athletes (*n* = 6), older endurance athletes (*n* = 4), and older control participants (*n* = 11). Blue color represents myosin heavy chain type I, green represents laminin, and orange represents NCAM-positive. ^b^Different from older control participants. One symbol indicates significance level of *P* ≤ 0.05. One-way ANOVA was used.

A higher percentage of NCAM-positive fibers coexpressing MHC I (main effect *P* = 0.038) was observed in OC (Fig. 6*C*) compared with OS (*P* = 0.047), OE (*P* = 0.039), and YC (*P* = 0.015). No difference was detected between OS, OE, and YC (*P* = 0.646–0.927).

NCAM-positive fibers were smaller than the mean fiber size in OC (3,340 ± 1,923 vs. 4,655 ± 1,464 µm; *P* = 0.020). No difference was detected in OE (2,888 ± 1,449 vs. 4,147 ± 247 µm; *P* = 0.216), OS (3,561 ± 2,665 vs. 4,542 ± 848 µm; *P* = 0.417), or YC (2,699 ± 1,792 vs. 4,218 ± 1,068 µm; *P* = 0.132).

## DISCUSSION

The main findings of the present study were that strength-trained master athletes had a type II fiber distribution similar to young individuals, and a higher proportion of type II fibers compared with endurance-trained master athletes and recreationally active older adults. Moreover, the strength-trained master athletes also displayed fewer signs of myocellular denervation, reflected by less prevalent myofiber grouping and fewer atrophic fibers compared with endurance-trained and recreationally active older adults, and again not being different from young. The present data imply that chronic strength training is an effective training modality for the maintenance of neuromuscular properties related to the recruitment of the fastest and largest motor units, in turn preserving type II fibers integrity, maximal muscle strength, and rapid force capacity at increasing age.

### Training Modality and Fast-Twitch Type II Fiber Properties

Fiber type distribution and grouping can be seen as the converging consequence of successive fiber denervation with or without subsequent reinnervation (12). A higher area-weighted proportion of type II fibers was observed in strength-trained master athletes compared with endurance-trained master athletes and recreationally active old. This appears to be explained by a combination of higher proportion of type IIa fibers and larger type II fiber area. Although evidence from studies investigating the effects of life-long strength compared with endurance training on type II fiber maintenance is scarce, our results are in accordance with previous results (27). Strikingly, type II fiber proportions and area-weighted distributions were highly similar between life-long strength-trained older adults (∼75 yr) and the young reference population (∼25 yr) recruited for the present study, both expressing a ∼50% type II fiber distribution typically expected in young (7).

### Training Modality and Fiber Type Grouping

Strength-trained master athletes were not different from young adults for any variable used to assess fiber type grouping. In contrast, older life-long endurance-trained master athletes and recreationally active older adults showed signs of type I fiber grouping, including larger fiber grouping size, higher fraction of grouped fibers, higher number of fiber type groups, and more atrophic fibers than young adults. Previous studies have failed to observe any differences in fiber type grouping between master athletes and older controls, which may be related to these studies collapsing endurance- and strength-trained master athletes into one group (11, 35). Thus, to our best knowledge, this is the first study to compare fiber type grouping in endurance- and strength-trained master athletes, which appears to be an important distinction. Different approaches to quantify fiber type grouping may also cause inconsistent results, as grouping may be overestimated even in young populations (35) where signs of fiber type grouping would not be expected (14, 39). Moreover, studies applying indirect neurophysiological estimates of motor unit numbers and size, an indicator of fiber reinnervation have left inconclusive evidence. Some observations suggest pronounced motor unit remodeling (increased motor unit size and/or low number of motor units) may take place in both strength- and endurance-trained master athletes compared with young (23, 40), whereas other studies have shown no motor unit remodeling in endurance-trained master athletes compared with recreationally active old (21, 22), suggesting a neuroprotective effect from endurance training. The current fiber grouping data derived from muscle biopsy analysis strengthen the notion that habitual recreational activity as well as high (life-long) endurance training volumes may offer similar benefits for fiber reinnervation with aging, but that they are of a different magnitude than strength training.

One likely explanation for the present signs of type I area-weighted dominance and fiber type grouping in endurance-trained master athletes and recreationally active older adults may be that the type II fibers are rarely used during repetitive contractions with low force requirement (41), which is typically the case in endurance exercise settings. When large, high-threshold motor units, critical for forceful and fast muscle contractions, become more inactive with age, their motor neurons may degrade. The resultant denervated myofibers, typically type II, may either be reinnervated by axon sprouting from adjacent motor neurons leading to myofiber type changes and myofiber grouping, or slowly waste away resulting in neurogenic atrophy, ultimately resulting in a loss of myofibers (12, 42). Although the low (absent) prevalence of fiber grouping in strength-trained master athletes could also potentially reflect denervation of fibers without subsequent reinnervation, this does not seem a plausible scenario due to these master athletes’ superior maximal strength, lack of fiber atrophy, high proportion of type II fibers, and enlarged thigh muscle volume, as also previously documented (26). Thus, although, we cannot exclude that the strength-trained master athletes had an inherent advantage compared with their aged-matched counterparts, our data are in line with observations in older adults performing high-load strength training, where increased type II muscle fiber area and distribution is observed (9, 43, 44). Interestingly, the differences observed between life-long strength-trained master athletes versus endurance-trained master athletes and untrained older adults in muscle morphology and fiber type grouping appear to perfectly mirror previous experimental observations on the neural side of the spectrum; namely that strength-trained master athletes demonstrate increased descending motor drive to maximally contracting skeletal muscle compared with endurance-trained, recreationally active and sedentary older adults (25, 26). Moreover, increased descending neural drive following short-term strength training has been documented in older adults (45–47). Collectively, these data suggest that strength training preserve neuromuscular activation to recruit the largest motor units innervating type II fibers, potentially due to performing recurrent high-force explosive-type motor activities dictated by their training protocols. This could be a key factor in maintaining the integrity of large-sized motor axons and for retaining type II fiber morphology at increasing age.

The present data may suggest that neither habitual physical activity nor high levels of long-term endurance training may be sufficient to maintain type II fiber properties and innervation with aging. Hence, despite higher volume of physical activity in the endurance-trained master athletes no differences in muscle fiber composition, grouping, or atrophic fibers were detected compared with recreationally active older adults, suggesting that the specific training modality and its pattern of muscle loading are of greater importance than training volume per se for type II fiber maintenance. However, it should be noted that the fiber type distribution patterns observed in recreationally active- and endurance-trained older adults could also reflect a training adaptation facilitating the high oxidative capacity associated with type I fibers. Yet, such oxidative myocellular characteristics would be expected to be more pronounced in the endurance-trained master athletes, which does not seem to be the case. Consequently, these patterns may instead reflect an aged phenotype.

### Training Modality and Markers of Denervation and Reinnervation

In line with the present fiber distribution and grouping data, life-long strength-trained master athletes demonstrated a lower density of atrophic fibers compared with endurance-trained master athletes and recreationally active old, with no difference compared with young controls. An abundance of atrophic fibers has previously been interpreted as poor reinnervation status as a consequence of progressive denervation (20, 36). Alternatively, an abundance of atrophic fibers could also reflect a lack of maximal voluntary motor unit recruitment, which similarly to denervation would result in a negative net protein balance in the affected fibers that would over time result in myofiber atrophy.

Somewhat surprisingly, a higher accumulation of muscle fibers with nuclear clumps was observed in strength-trained and recreationally active older compared with young controls in the present study, suggesting the presence of more fibers with severe neurogenic atrophy in these aging groups (19). These data may suggest some level of complete myofiber denervation in strength-trained athletes and recreationally active aged, which may in turn suggest superior reinnervation in the endurance-trained. However, fibers with nuclear clumps were detected only in few individuals (8/42), and in a very low number of affected fibers (mean: 2–4‰) making it difficult to draw any firm conclusion about the functional relevance of these findings. It should be noted that previous studies have reported fibers with nuclear clumps to be observed only in frail older adults (11), and of higher magnitude (∼2%) than observed in the present study, suggesting that the groups examined in the present study were representative of healthy, active aging without severe atrophy.

NCAM-positive fibers did not differ between age groups or training modes in the present study. Elevated numbers of NCAM-positive fibers have previously been reported in older adults compared with young, even in life-long trained athletes (17, 48). Similar to the present data Sonjak et al. (11), did not observe differences between frail older adults, age-matched master athletes, and young controls. Notably, NCAM has been shown to be present during both denervation and reinnervation (16) and seem not to be constantly expressed in denervated muscle (49), which in combination with the inclusion of healthy individuals may explain the low proportion of NCAM-positive fibers (1.0–7.4‰) and thus lack of age-related differences in NCAM-positive fibers. Furthermore, NCAM-positive fibers may also be observed in conditions not related to fiber denervation/reinnervation, such as exercise-induced regeneration and myopathy (50, 51), which challenges the use of NCAM as a 1:1 marker of denervation.

As all markers are not continuously expressed during muscle fiber denervation, we used a combination of markers of myofiber grouping to indicate long-term denervation with subsequent reinnervation (13, 35), NCAM-positive fibers to indicate acute denervation (17, 48), and pyknotic nuclei bags and atrophic fibers (very small fibers) to indicate long-term denervation without subsequent reinnervation (11, 19, 20, 36). These markers were chosen to represent the denervation status in a population that was regarded as healthy, where denervation had likely not progressed to a very high level. A combination of different markers such as that used in the present study has also previously been utilized (11).

### Influence of Training Modality on Muscle Strength and Rate of Force Development

From a functional perspective, it is of interest that the strength-trained master athletes demonstrated ∼30%–294% higher maximal strength and RFD than their age-matched counterparts. This observation is in line with previous reports from our laboratories comparing life-long strength-trained master athletes to life-long endurance-trained master athletes and untrained/recreationally active older adults (25–27). The present endurance-trained master athletes also demonstrated higher levels of maximal lower limb muscle strength and RFD compared with recreationally active old, suggesting a degree of preserved neuromuscular function in the endurance-trained group. The ability to produce force rapidly, that is, as much force as possible in a short time frame, is particularly important in for example, fall prevention, given that during tripping or loss of postural balance there is only limited time to regain balance (52). As it typically takes more than 300 ms to reach maximum muscle force (53, 54), RFD is considered to be more functionally relevant than maximal muscle strength (55), especially at older age (56). The higher RFD demonstrated in strength-trained master athletes may be related, at least in part, to the previously documented higher descending drive (26) and higher proportion of type II fibers, both of which have been associated to greater RFD (55, 57–60). Although, the relationship between higher intrinsic single fiber RFD and joint-level RFD has not been firmly established (58). Moreover, other factors not examined in the presented study also influence RFD, such as tendon stiffness and moment arm/lever length (55).

### Methodological Considerations

Although the present data suggest a protective effect of life-long strength training in preserving neuromuscular function related to strong and rapid muscle contractions, it is not possible to exclude a role of potential confounding factors including genetics, nutritional intake, and exposure/absence to various stressors. Moreover, the cross-sectional study design also impedes firm conclusions regarding the evolution of fiber type composition. Further, fiber grouping in the present study was defined as phenotype-specific groups of fibers with one or more enclosed fibers. Of notice, the likelihood of observing enclosed fibers is higher if the individual has a dominance of one fiber type over the other. To account for this, we presented these variables expressed both as a percentage of total fiber count as well as percentage of type I fibers. The low number of participants in each group challenges definitive conclusions and the present findings should therefore be interpreted with caution, although the relatively small groups are explained by the low prevalence of master athletes in the general population. It should also be noted that these results may not extend to other forms of endurance training, such as cycling, rowing, and skiing. Finally, although we would also preferably have included females in the present study, this was not possible as we were unable to identify female strength master athletes in the local area, thus limiting the participant cohort to males.

### Conclusions

Life-long strength-trained master athletes demonstrate neuromuscular properties related to fast-twitch type II fiber force production that are of comparable magnitude to young recreationally active individuals. Similarly, life-long endurance-trained master athletes and recreationally active old demonstrate similar neuromuscular properties, with lower proportion of type II fibers and more markers of grouping. Consequently, strength training appears to counteract age-related denervation processes and concurrent atrophy of type II fibers in older men to thereby promote maintenance of maximal strength and RFD, which is critically important for retaining functional capacity with increasing age.

## DATA AVAILABILITY

Data are available upon reasonable request to the corresponding author.

## GRANTS

Funding for this project was received from Molde University College.

## DISCLOSURES

No conflicts of interest, financial or otherwise, are declared by the authors.

## AUTHOR CONTRIBUTIONS

T.T., O.K.B., U.F., P.A., and E.W. conceived and designed research; T.T., O.K.B., M.F.B., S.K.N., L.E., T.M., and E.W. performed experiments; T.T., J.L.N., O.K.B., and E.W. analyzed data; T.T., J.L.N., O.K.B., M.F.B., P.A., and E.W. interpreted results of experiments; T.T. prepared figures; T.T. and J.L.N. drafted manuscript; T.T., J.L.N., O.K.B., M.F.B., S.K.N., L.E., T.M., P.A., and E.W. edited and revised manuscript; T.T., J.L.N., O.K.B., M.F.B., S.K.N., L.E., T.M., P.A., and E.W. approved final version of manuscript. 
